# Mental health and sleep correlates of self-reported outdoor daylight exposure in over 13,000 adults with depression

**DOI:** 10.1192/j.eurpsy.2025.20

**Published:** 2025-02-17

**Authors:** Jacob J. Crouse, Shin Ho Park, Brittany L. Mitchell, Enda M. Byrne, Sarah E. Medland, Tian Lin, Jan Scott, Zsofi de Haan, Emiliana Tonini, Frank Iorfino, Naomi R. Wray, Nicholas G. Martin, Ian B. Hickie

**Affiliations:** 1 Youth Mental Health and Technology Team, Brain and Mind Centre, The University of Sydney, NSW, Australia; 2 Mental Health and Neuroscience Program, QIMR Berghofer Medical Research Institute, Brisbane, QLD, Australia; 3 Institute for Molecular Bioscience, The University of Queensland, Brisbane, QLD, Australia; 4 Child Health Research Centre, The University of Queensland, Brisbane, QLD, Australia; 5 Academic Psychiatry, Institute of Neuroscience, Newcastle University, Newcastle upon Tyne, UK; 6 Queensland Brain Institute, The University of Queensland, Brisbane, QLD, Australia; 7 Department of Psychiatry, The University of Oxford, Oxford, UK; 8 Oxford Big Data Institute, Li Ka Shing Centre for Health Information and Discovery, University of Oxford, Oxford, UK

**Keywords:** daylight, sunlight, insomnia, circadian, chronobiology, mood disorders

## Abstract

**Background:**

Increasing daylight exposure might be a simple way to improve mental health. However, little is known about daylight-symptom associations in depressive disorders.

**Methods:**

In a subset of the Australian Genetics of Depression Study (*N* = 13,480; 75% female), we explored associations between self-reported number of hours spent in daylight on a typical workday and free day and seven symptom dimensions: depressive (overall, somatic, psychological); hypo-manic-like; psychotic-like; insomnia; and daytime sleepiness. Polygenic scores for major depressive disorder (MDD); bipolar disorder (BD); and schizophrenia (SCZ) were calculated. Models were adjusted for age, sex, shift work status, employment status, season, and educational attainment. Exploratory analyses examined age-stratified associations (18–24 years; 25–34 years; 35–64 years; 65 and older). Bonferroni-corrected associations (*p* < 0.004) are discussed.

**Results:**

Adults with depression reported spending a median of one hour in daylight on workdays and three hours on free days. More daylight exposure on workdays and free days was associated with lower depressive (overall, psychological, somatic) and insomnia symptoms (*p*’s<0.001), but higher hypo-manic-like symptoms (*p*’s<0.002). Genetic loading for MDD and SCZ were associated with less daylight exposure in unadjusted correlational analyses (effect sizes were not meaningful). Exploratory analyses revealed age-related heterogeneity. Among 18–24-year-olds, no symptom dimensions were associated with daylight. By contrast, for the older age groups, there was a pattern of more daylight exposure and lower insomnia symptoms (*p* < 0.003) (except for 25–34-year-olds on free days, *p* = 0.019); and lower depressive symptoms with more daylight on free days, and to some extent workdays (depending on the age-group).

**Conclusions:**

Exploration of the causal status of daylight in depression is warranted.

## Introduction

Mental disorders are associated at multiple levels with circadian disruption [1–4], and it has been suggested that robust circadian rhythmicity is essential for supporting optimal mental health [5–7]. In humans, a central “circadian” (~24-hour) clock in the brain regulates the timing of most physiological processes and behaviours including mood, energy, activity, and cognition [8–10]. Daily exposure to sunlight—a free, readily available resource—is the primary input that regulates circadian rhythms and their alignment to the day-night cycle [11–17]. Circadian rhythms evolved over millions of years under a relatively stable pattern of bright light during the day and limited light at night [18]. Industrialisation led to the widespread use of artificial indoor lighting, which is too dim during the day for optimal entrainment [19]. The suboptimal intensity and patterns of typical artificial light exposure during the day, and the fact that industrialised societies spend ~90% of their time indoors and only ~2.5 hours in daylight per day [20,21], is a major diversion in the light patterns under which our circadian system evolved. Ultimately, this challenges the stability of circadian rhythms, which may impact mental health conditions including depressive disorders [22].

Greater exposure to daylight appears to have a positive effect on mental health in the general population [23–25]. Two studies performed using UK Biobank (*N* = 85,000–400,000) reported that higher self-rated and objective daylight exposure were associated with lower levels of low mood, anhedonia, antidepressant usage, and major depressive disorder (controlling for nighttime light exposure, photoperiod, sleep quality, sociodemographic factors, physical activity, and cardio-metabolic health) [20,26]. Some community-based studies report opposing findings. One study exploring the effects of sunlight exposure in a community sample reported a lack of association between sunlight and depressive symptoms [27], and a study of >13,000 university graduates reported that males living in locations with longer daylight hours had a higher (not lower) risk of depression [28]. Several small clinical studies suggest a beneficial pattern of association. In subjects with seasonal affective disorder (SAD), those allocated to a 1-hour morning natural light therapy (walking outdoors) reported a larger reduction in depressive symptoms compared to those allocated to daily artificial light therapy [29]. And a meteorological study of people with SAD observed that shorter daylight duration and lower daylight exposure were associated with worse depressive symptoms a week later [30].

Two gaps are worth noting. First, most studies exploring the daylight-depression relationship focus narrowly on sum scores from depressive symptom scales, which usually combine psychological and somatic/physical symptoms. These may represent separate dimensions [31]. To the best of our knowledge, only one study has examined specific depressive symptom types, finding that participants exposed to more sunlight were less likely to report suicidal thoughts, and more likely to report changes in sleep, appetite, and feelings of worthlessness/guilt [32]. Second, studies have neglected other symptom dimensions experienced by people with depression, including psychotic-like phenomena and hypo/mania, which might be influenced by light, as in bipolar disorder [33–35]. Examining a link between daylight and hypo/manic symptoms is important, as at lower levels of severity, hypo/mania may actually reflect *positive* states of increased mood and energy, rather than psychopathology.

Using a volunteer cohort of adults with depression (*Australian Genetics of Depression Study*), our objective was to explore the associations between self-reported number of hours of outdoor natural light exposure (on workdays and free days) and seven symptom dimensions. Given this cohort is genetically informative, we also explored whether genetic vulnerability to mood and psychotic disorders is associated with lower levels of daylight exposure. We hypothesised that more hours of self-reported daylight exposure would be associated with lower levels of depressive symptoms (overall, psychological, somatic dimensions), insomnia symptoms, daytime sleepiness, and psychotic-like experiences, but higher levels of increased activity and mood (i.e., sub-threshold hypo-mania-like phenomena). Examination of differences in light-symptom associations on workdays vs free days was exploratory. Our analyses and hypotheses were not pre-registered.

## Methods

### Participants and study design

The Australian Genetics of Depression Study (AGDS) is a volunteer cohort of adults diagnosed with, and/or treated for, a depressive disorder. Recruitment procedures and sample characteristics are described elsewhere [36]. Participants joined AGDS after responding to a media campaign or a letter from the Australian Government’s Department of Human Services, mailed to 110,000 Australian residents who had received four or more antidepressant prescriptions for any of the 10 most common antidepressants in Australia, over the prior 4.5 years.

Participants completed an online survey about physical and mental health, treatment for depression, and factors related to mental health [36]. Participants could donate a saliva sample for genetic analyses; >75% returned a sample via mail-out kit [37].

Sources of bias in AGDS include: (a) high educational attainment; (b) recruited mostly via media appeal (>85%) compared to prescription history invitation; (c) higher female-to-male ratio than population prevalence of unipolar depression (3:1 vs 2:1); and (d) highly recurrent course of depression (modal number of episodes = 13+) [36].

The study received ethical approval from the QIMR Berghofer Medical Research Institute Human Research Ethics Committee in Brisbane, Australia. Written informed consent was obtained.

### Assessments

#### Demographics

Age, biological sex, marital status, and educational attainment were collected using standard questions.

#### Daylight exposure

Participants were asked: “On average, how much time do you spend outdoors in natural light per day?”, separately for workdays and free days (e.g., weekend). Responses were in hours and minutes. We employed data cleaning rules to remove implausible values: (a) values like ‘360 hours’ (meant to be ‘360 minutes’); and (b) values greater than the maximum hours of daylight on the longest day of the year (15 hours). 17 and 28 values were deleted for workdays and free days, respectively (<0.001% of data).

#### Depressive symptoms

The 12-item Somatic and Psychological Health Report (SPHERE-12) [38] assessed somatic and psychological symptoms of depression. Participants responded to items with the prompt, “*Over the past few weeks have you been troubled by*…” Responses were coded: “Never or some of the time” (=1); “A good part of the time” (=2); and “Most of the time” (=3). We used a total score and sub-scale scores for the somatic (SOMA-6) and psychological dimensions (PSYCH-6).

#### Hypo/manic-like symptoms

A 5-item screener adapted from the Altman Self-Rating Mania Scale (ASRMS) [39] assessed lifetime, brief periods of hypo-manic-like symptoms. Participants responded to items with the prompt, “*Have you ever experienced a definite period where for more than 2 or 3 days*…” Responses were coded: ‘Don’t know’ (=0), ‘No’ (=0), and ‘Yes’ (=1). We used a total score (range=0-5).

#### Psychotic-like symptoms

A 6-item screener partially adapted from the Community Assessment of Psychic Experiences (CAPE) [40] assessed lifetime psychotic-like symptoms. Participants responded to items with the prompt, “*Have you ever*…” Responses were coded: “No” (=0) and “Yes” (=1). We used a total score (range = 0–6).

#### Insomnia symptoms

The 7-item Insomnia Severity Index (ISI) [41] assessed current (last 2 weeks) severity of insomnia symptoms. We used a total score (range = 0–28).

#### Daytime sleepiness

The 8-item Epworth Sleepiness Scale (ESS) [42] assessed excessive daytime sleepiness. Participants responded to a prompt about their likelihood of dozing in eight situations. Responses were coded: ‘Would never doze’ (=0); “Slight chance of dozing” (=1); ‘Moderate chance of dozing’ (=2); and ‘High chance of dozing’ (=3). We used a total score (range = 0–24).

#### Employment and shift work

Participants were asked “How many days do you work on average?” and we coded an employment status variable as: “full-time” (4–7 days); “part-time” (1–3 days); and “not working” (0 days). Participants were asked “Which of the following best describes your current work arrangements?” and we coded a “Shift work status” variable as: “shift-worker” (1 = shift work with rotating shifts of irregular shifts; 0 = regular work schedule).

### Polygenic risk scores (PGS)

Genotyping was conducted on the Illumina Global Screening Array V2. Samples were merged with the 1000 Genomes [43] project samples and genetic principal components were calculated using a set of single nucleotide polymorphisms (SNPs) not in linkage disequilibrium. Pre-imputation quality control was done using PLINK 1.9 [44,45], including removing SNPs with a minor allele frequency <0.005, SNP call rate <97.5%, and identification of participants with genetic similarity to a European reference group (>4 SD from Ancestry Principal Components [PCs] PC1/PC2 centroid) [46] and Hardy–Weinberg equilibrium (*p* < 1 × 10^−6^), before imputation using the Haplotype Reference Consortium 1.1 reference panel [47]. Over 95% of AGDS is of European ancestry and PGS were created only for those of European ancestry. Summary statistics from recent genome-wide association studies (GWAS) were used to create PGS for major depressive disorder (MDD) [48], bipolar disorder (BD) [49], and schizophrenia (SCZ) [50]. SBayesRc [51], a Bayesian PGS method, was used to generate allele weights for each PGS. The posterior SNP effects for each disorder were used to generate PGS for each participant using the PLINK – score function [44]. As some AGDS participants have been included in depression GWAS [48], the depression-PGS was calculated using summary statistics excluding AGDS participants.

### Statistical analysis

Analyses were conducted using R (version 4.2.2) [52]. The sample size was not pre-determined for this analysis. We included all participants with data about light exposure and any mental health and sleep data. Before analysis, we normalised the variables of the reported hours of daylight exposure on free days and workdays, given their positive skew. Correlations among hours of daylight exposure on workdays and free days and MDD, BD, and SCZ PGS were examined using Spearman correlations (rho). Fourteen regression models were used to examine the associations among the seven symptom total or sub-scale scores (SPHERE-12; PSYCH-6; SOMA-6; ASRMS; CAPE; ISI; ESS) and the self-reported hours of daylight exposure on workdays and free days. We ran separate regression models for workdays and free days given their moderate correlation (*r* = 0.47). Models were adjusted for relevant confounders (age, sex, employment status, shift worker status, and season). We reserve the use of ‘significant’ for associations with a Bonferroni-corrected *p* < 0.004 (0.05/14).

## Results

The analytic sample included up to 13,480 participants (75% female, mean age = 43.1 years; Table 1). The sample is highly educated (28% have a postgraduate degree, 36% have a degree, and 87% have a post-high school qualification). As reported previously [36], these rates are higher than other Australian general population control samples such as QSkin (56% with a post-high school qualification).

**Table 1.** Socio-demographic characteristics of the sample (*N* = 13,480)

Variable	Mean (SD) or *N* (%)	Range
Age (years)	43.08 (15.30)	18–89
Sex (female)	10,049 (74.60)	—
Marital status		
Married or de facto relationship	7,125 (52.86)	—
Separated or divorced	1,982 (14.70)	—
Widowed	214 (1.59)	—
Never married	4,137 (30.69)	—
Not provided	22 (0.16)	—
Educational attainment		
Postgraduate degree	3,722 (27.61)	—
Degree	4,813 (35.70)	—
Certificate or diploma	3,134 (23.25)	—
Senior high school or less	1,062 (7.88)	—
Junior high school or less	739 (5.48)	—
No formal education (or not provided)	10 (0.07)	—
Employment status		
Full-time work (4–7 days)	7,878 (61.22)	—
Part-time work (1–3 days)	2,374 (18.45)	—
Not working (0 days)	2,616 (20.33)	—
Shift worker status	1,569 (11.64)	—

**Figure 1.**
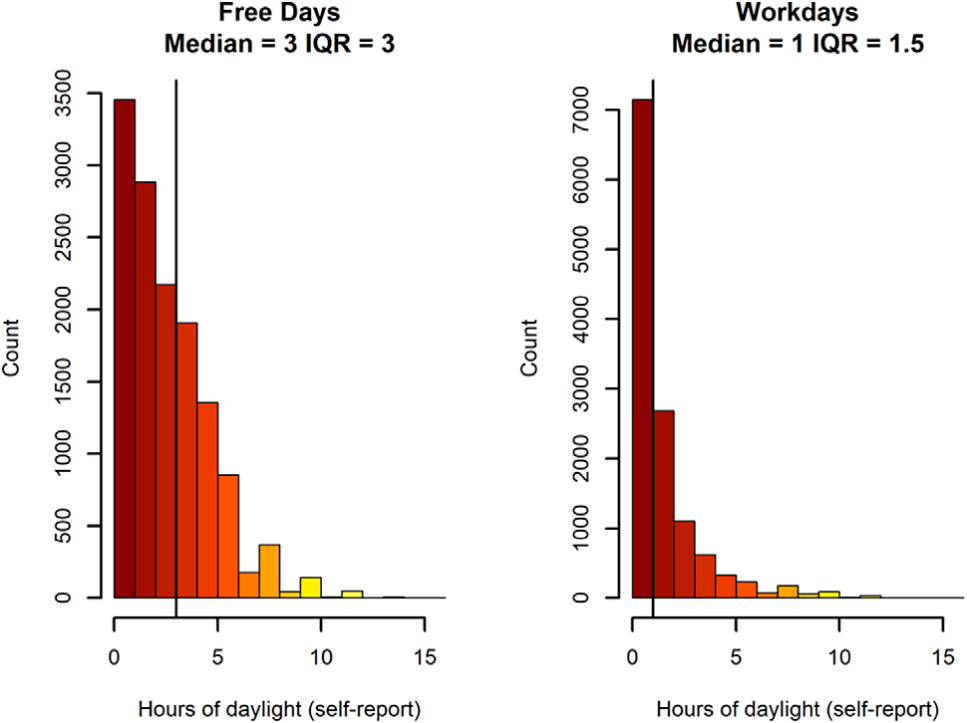
Self-reported hours of natural daylight exposure on free days and workdays in adults with depression (*N* = 13,480). *Note*: black vertical line represents the median.

Most met the criteria for a DSM-5 lifetime major depressive episode (Table 2). Depressive symptoms were in the low-to-moderate range (SPHERE-12; mean = 8.85); 28.2% had suggestive clinical insomnia of moderate severity (ISI = 15–21), 6.2% had suggestive severe clinical insomnia (ISS = 22–28); and 3.4% had excessive sleepiness (ESS = 16–24). The sample reported a median of 3 hours of outdoor light exposure on free days (IQR = 3; range = 0–14) and a median of 1 hour on workdays (IQR = 1.5; range = 0–14) (Figure 1).

**Table 2.** Mental health and sleep characteristics of the sample (N = 13,480)

Variable	Mean (SD) or *N* (%)	Range
Major depressive episode (DSM–5)	11,924 (88.46)	—
Depressive symptoms (SPHERE–12)	8.85 (6.50)	0–24
Psychological symptoms (PSYCH–6)	4.12 (3.72)	0–12
Somatic symptoms (SOMA–6)	4.73 (3.51)	0–12
Psychotic-like symptoms (CAPE; abbrev.)	0.91 (1.37)	0–6
Hypo-manic-like symptoms (ASRMS; abbrev.)	2.62 (2.08)	0–5
Insomnia symptoms (ISI)	12.70 (5.23)	1–28
Daytime sleepiness (ESS)	6.51 (4.23)	0–24

*Note*: DSM-5=Diagnostic and Statistical Manual of Mental Disorders (version 5); SPHERE-12=Somatic and Psychological Health Report; CAPE=Community Assessment of Psychic Experiences; ASRMS=Altman Self-Rating Mania Scale; ISI=Insomnia Severity Index; ESS=Epworth Sleepiness Scale.

We explored whether three PGS (which we would expect to be associated with the symptom measures) were associated with daylight exposure. Neither the MDD, BD, or SCZ PGS were associated with daylight exposure on workdays (correlation coefficients <0.05); however, there were very small but significant associations between fewer hours of daylight exposure on free days and higher PGS for MDD (rho = −0.03; *p* = 0.019) and SCZ (rho = −0.03; *p* = 0.006). Given the size of these correlations, we elected to exclude the PGS as covariates in the regression models.

### Light exposure and mental and sleep health

**Figure 2.**
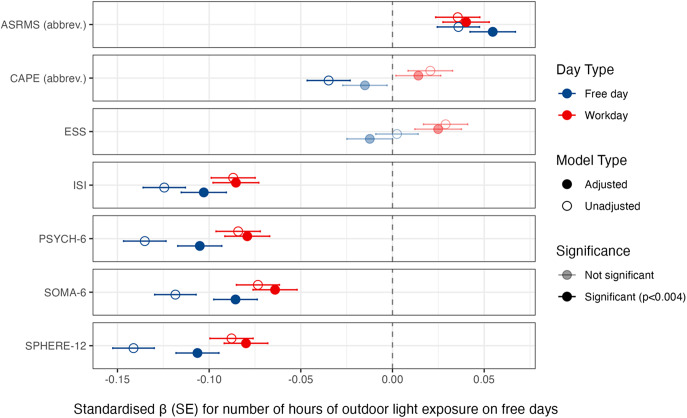
Self-reported number of hours of daylight exposure on free days and workdays and dimensions of mental and sleep health. *Note*: Coefficients to the left of the broken line indicate that more outdoor daylight exposure is associated with a lower level of symptoms. Adjusted models include covariates of age, sex, shift worker status, employment status, educational attainment, and season. SPHERE-12 = Somatic and Psychological Health Report; CAPE = Community Assessment of Psychic Experiences; ASRMS = Altman Self-Rating Mania Scale; ISI = Insomnia Severity Index; ESS = Epworth Sleepiness Scale.

In Figure 2, we summarise the results of the regression models exploring associations among symptoms and self-reported daylight exposure on workdays and free days.

On workdays (adjusting for covariates), more self-reported hours of daylight exposure was associated with lower overall depressive symptoms (SPHERE-12; *β* = −0.08; *p* < 1.94 × 10^−11^); lower somatic symptoms (SOMA-6; *β* = −0.06; *p* < 1.00 × 10^−7^); lower psychological symptoms (PSYCH-6; *β* = −0.08; *p* < 9.65 × 10^−11^); lower insomnia symptoms (ISI; *β* = −0.09; *p* < 8.15 × 10^−12^); and higher hypo-mania-like symptoms (ASRMS; *β* = 0.04; *p* = 0.001). Associations with psychotic-like symptoms (CAPE; *β* = 0.01; *p* = 0.250) and excessive daytime sleepiness (ESS; *β* = 0.02; *p* = 0.050) were not significant. Age was associated with all seven outcomes (*p*’s < 0.008), with increasing age associated with higher daytime sleepiness (ESS), but lower symptoms on the remaining six scales. Model outputs are in the Supplementary Materials.

On free days (adjusting for covariates), more self-reported hours of daylight exposure was associated with lower overall depressive symptoms (SPHERE-12; *β* = −0.11; *p* < 1.19 × 10^−19^); lower somatic symptoms (SOMA-6; *β* = −0.09; *p* < 5.61 × 10^−13^); lower psychological symptoms (PSYCH-6; *β* = −0.11; *p* < 2.50 × 10^−18^); lower insomnia symptoms (ISI; *β* = −0.10; *p* < 7.24 × 10^−17^); and higher hypo-mania-like symptoms (ASRMS; *β* = 0.05; *p* < 1.01 × 10^−5^). Associations with psychotic-like symptoms (CAPE; *β* = −0.02; *p* = 0.214) and daytime sleepiness were not significant (ESS; *β* = −0.01; *p* = 0.322). Age was associated with all seven outcomes (*p*’s < 0.002), and in the same direction as the workday models. Model outputs are in the Supplementary Materials.

### Exploratory analyses

Given the associations between symptoms and age (Spearman’s *r* = −0.11 to −0.26) and the small correlations between age and self-reported light exposure on workdays and free days (rho = 0.09–0.12), we conducted exploratory analyses of light-symptom associations by age-group. We used categories commonly used in national and epidemiologic studies (e.g., Australian Bureau of Statistics, Australian Institute of Health and Welfare) [53,54]: 18–24 years; 25–34 years; 35–64 years; and 65+. We re-examined associations between PGS for depression, schizophrenia, and bipolar disorder within each age group; correlation coefficients were small: 18–24 years (rho = −0.03 to 0.02); 25–34 years (rho = −0.05 to −0.01); 35–64 years (rho = −0.05 to −0.01); and 65+ (rho = 0–0.03). We elected to exclude them as covariates in the age-stratified models. Table 3 summarises the light exposure, sleep, and mental health ratings across the age groups.

**Table 3.** Differences in light exposure and mental health and sleep ratings across age groups

	18–24 years (*N* = 1,636)	25–34 years (*N* = 3,242)	35–64 years (*N* = 7,301)	65-and-older (*N* = 1,301)
Variable	Mean (SD)			
Light (free days)	2.75 (2.20)	2.84 (2.04)	3.14 (2.18)	3.31 (2.16)
Light (workdays)	1.83 (1.79)	1.54 (1.70)	1.81 (1.91)	2.61 (2.09)
Depressive symptoms (SPHERE–12)	11.60 (6.37)	10.00 (6.45)	8.26 (6.37)	5.78 (5.53)
Psychological symptoms (PSYCH–6)	5.61 (3.71)	4.83 (3.69)	3.78 (3.65)	2.27 (3.03)
Somatic symptoms (SOMA–6)	5.99 (3.46)	5.16 (3.48)	4.47 (3.48)	3.52 (3.20)
Psychotic-like symptoms (CAPE; abbrev.)	1.51 (1.68)	1.04 (1.42)	0.80 (1.28)	0.46 (0.92)
Hypo-manic-like symptoms (ASRMS; abbrev.)	3.06 (1.96)	2.76 (2.11)	2.49 (2.10)	2.49 (1.94)
Insomnia symptoms (ISI)	13.42 (5.07)	12.96 (5.15)	12.60 (5.30)	11.41 (4.91)
Daytime sleepiness (ESS)	6.49 (4.20)	6.41 (4.08)	6.59 (4.30)	6.37 (4.24)

**Figure 3.**
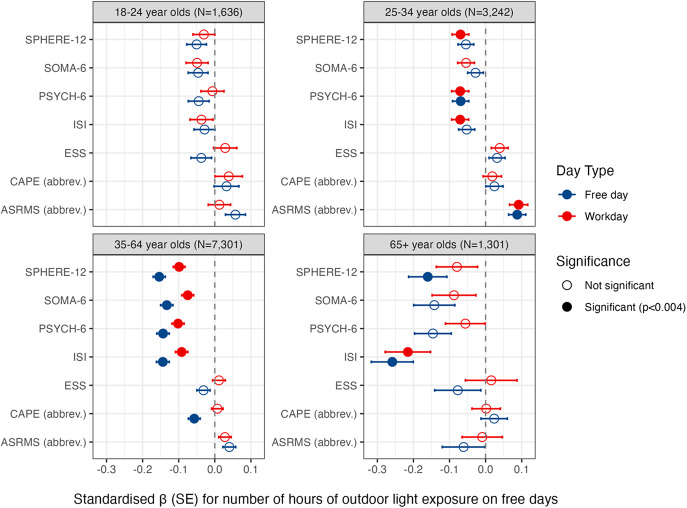
Exploratory analyses of self-reported hours of daylight exposure on free days and workdays and sleep and mental health across age-groups. *Note*: Models are adjusted for age, sex, shift worker status, employment status, educational attainment, and season. We illustrate unadjusted associations in Supplementary Figure 2. SPHERE-12 = Somatic and Psychological Health Report; CAPE = Community Assessment of Psychic Experiences; ASRMS = Altman Self-Rating Mania Scale; ISI = Insomnia Severity Index; ESS=Epworth Sleepiness Scale.

Age-stratified analyses revealed age-related heterogeneity (Figure 3). For the youngest group (18–24 years; *N* = 1,636), there were no significant associations between any symptom measure and light exposure on workdays or free days. For the three older age groups, there was a generally consistent association of lower insomnia symptoms (ISI) and more hours of daylight exposure on workdays and free days; but not for the 25–35-year-olds on free days. Similarly, there was a generally consistent association of lower overall depressive symptoms (SPHERE-12) and more hours of daylight exposure on free days for the two groups aged 35+; and to a lesser extent on workdays, which was significant for only the two groups aged 25–64. Model outputs are in the Supplementary Materials.

### Sensitivity analysis

Finally, we explored whether the associations between daylight exposure and sleep and mental health were robust to adjustment for self-rated chronotype and insomnia (two variables associated with daylight exposure and mental health [55–59]). After adding the reduced Morningness-Eveningness Questionnaire (rMEQ) total score and the ISI to separate regression models, we found that the patterns of association were similar, with many of the previously significant associations remaining nominally significant (*p* < 0.05), albeit attenuated in magnitude. The general pattern of more daylight on workdays and free days with lower depressive symptoms (SPHERE-12, SOMA-6, PSYCH-6) and higher hypo-mania-like symptoms was robust to these adjustments in analyses of the whole sample (Supplementary Figures 3–4). In the age-stratified analyses, the pattern of more daylight on workdays and free days with lower depressive symptoms (SPHERE-12, SOMA-6, PSYCH-6) was robust to adjustments in the 35–64-year-old group, while adjusting for ISI had a more significant attenuating influence in the 25–34-year-olds and 65+ year olds (Supplementary Figures 5–6).

## Discussion

In an Australian volunteer cohort of people with depression, adults aged 25+ who report spending more time in natural light on a typical day generally reported lower levels of recent depressive and insomnia symptoms. Alongside other studies [17,23], our findings support a link between daylight and depressive phenomena. Sleepiness and psychosis-like symptoms were largely unrelated to daylight exposure. The results for hypo-mania-like symptoms and somatic and psychological depressive symptoms varied more strongly according to age group. Before discussing these findings, several limitations are worth mentioning.

First, exposure to natural light was self-reported using a single-item question that is less comprehensive than other scales (e.g., *Harvard Light Exposure Assessment* [60]). Relatedly, differences in built environments (e.g., window access) may cause differences in *indoor* daylight exposure between people reporting the same hours of *outdoor* exposure. Furthermore, the timing of daylight and nocturnal light exposure were not ascertained, which matters given the associations between both day-time [26] and night-time light exposure and depression [26,61]. Third, although assessments of mental health, sleep, and daylight exposure were completed concurrently, our data lacked temporal information. Inferences about directionality and causality are not possible, especially given that these states (and light exposure) are dynamic [62]. Fourth, the strength of the PGSs is reliant on the strength of the GWAS, and the PGS may be underpowered to detect the effect on light exposure. Fifth, this cohort is biased toward high educational attainment, which is associated with mental and sleep health and time spent indoors [63]. Associations between daylight exposure and mental health and sleep may be affected by collider bias induced by the selection of high educational attainment [64]. The magnitude of this is unknown, but we note that coefficients were similar when including/excluding educational attainment as a covariate (Supplementary Figure 1). Sixth, there are risks associated with overexposure to ultraviolet light. Recommendations about increasing daylight exposure for the benefit of sleep and mental health should also communicate how to safeguard potential harms. Finally, possible confounders such as physical activity and day length at the time of assessment were unavailable, and while data on *lifetime* medications was recorded, data on *current* medications which may increase (e.g., citalopram) [65] or decrease light sensitivity (e.g., lithium) [35] were unavailable. We now discuss interpretations of these findings and areas for innovation.

As hypothesised, the associations between time in daylight and mental/sleep health largely indicated a relationship between better mental/sleep health with more time in daylight. This was clearest for insomnia and depressive symptoms (Figures 2 and 3). Associations were generally stronger on free days, wherein people have more control over how they spend their time. Our first interpretation of these associations is a causal effect of daylight on symptoms. There may be a beneficial effect of light on the neural regulation of mood circuits, as observed in animals whereby light information is signalled via photosensitive retinal ganglion cells to neurons in the perihabenular hub of the thalamus that are purported to control mood-related behaviours [66–70]. There may also be a beneficial effect of daylight on the entrainment and stabilisation of circadian rhythms, which regulate mood, energy, and sleep [71,72]. An alternative interpretation is noncausal effect, whereby opportunities for behaviours benefiting mood/sleep (e.g., socialising, exercise) [73] may occur more in daylight. Conversely, these associations might be explained by an effect of symptoms on daylight: people with depression may be more likely to stay indoors when depressed or sleeping poorly [74]. Finally, low daylight exposure and symptoms might be caused by common genetic or unmeasured factors (e.g., sedentary behaviour). These questions might be clarified by applying causal inference methods to longitudinal designs that measure dynamic mental states and objective recordings of sleep, activity, and light [75].

One noteworthy (exploratory) finding was the age-related differences in light exposure and light-symptom associations. On free days, the 18–24-year and 25–34-year age groups reported similar hours of light exposure, while the 35-64-year and 65+ groups reported more hours of daylight (Table 3). By contrast, on workdays, the 65+ age group reported more daylight exposure than the 18–24-year, 25–34-year, and 35–64-year groups. One study reported that although all age groups spend more than half of their waking hours in dim or moderate room light intensity, older adults spend more of their waking day in brighter light [76]. In the three older groups, there were stronger associations between depressive and insomnia symptoms and daylight exposure on workdays and free days, with some slight differences depending on the specific association (Figure 3). By contrast, daylight and symptoms were unrelated in the 18–24-year-olds. One interpretation is that, if a causal effect of daylight exposure on symptoms is real, it may be less relevant to youth, for whom psychopathology may be more strongly influenced by other factors (e.g., stressors around the transition to adulthood; academic pressure; bullying; neurobiological maturation; sensitivity to social influence) [77–79]. By contrast, the mental health and sleep of individuals in the older age-groups may be more strongly dependent on light exposure or activities correlated with time spent outdoors (e.g., socialising, exercise).

Another noteworthy finding was that more daylight exposure was associated with *higher* lifetime hypo-mania-like symptoms. A simple interpretation is that some symptoms (e.g., increased activity) may cause people to spend more time outdoors. An alternative interpretation is that the items used are based on a screening tool that does not ask about severity/impairment, and as such, these items may be indexing *positive* mental states related to being outside (e.g., being active, feeling elated, increased self-confidence) rather than psychopathology.

In conclusion, we observed that in adults with depression (and particularly those aged 25 and older), more reported time spent in daylight is associated with better mental and sleep health, and especially depressive and insomnia symptoms. Exploration of the causal status of daylight on mental and sleep states is warranted [80]. Better estimation of light exposure using wearables may provide novel insights.

## Supporting information

Crouse et al. supplementary material 1 Crouse et al. supplementary material

Crouse et al. supplementary material 2 Crouse et al. supplementary material

## Data Availability

Access to AGDS data is restricted due to the ethical guidelines governing the study but may be accessible following ethical review and data transfer agreements. Please contact Nicholas Martin (nick.martin@qimrberghofer.edu.au) with queries related to accessing AGDS data.

## Supplementary material

The supplementary material for this article can be found at http://doi.org/10.1192/j.eurpsy.2025.20.
