# Supplementary material for: Mental health and sleep correlates of self-reported outdoor daylight exposure in over 13,000 adults with depression
Source: Eur Psychiatry. 2025 Feb 17;68(1):e41. doi: 10.1192/j.eurpsy.2025.20 (PMC12041733; doi:10.1192/j.eurpsy.2025.20)
Supplement: Crouse et al. supplementary material 2 — Crouse et al. supplementary material [file S0924933825000203sup002.docx]

**Supplementary Figure 1. Summary of unadjusted and two versions of adjusted regression models (with and without education) examining associations among self-reported number of hours of outdoor light exposure on workdays and free days and mental and sleep health.** Note**:** Coefficients to the left of the broken line indicate that a greater number of hours of outdoor light exposure is associated with lower level of each symptom dimension.


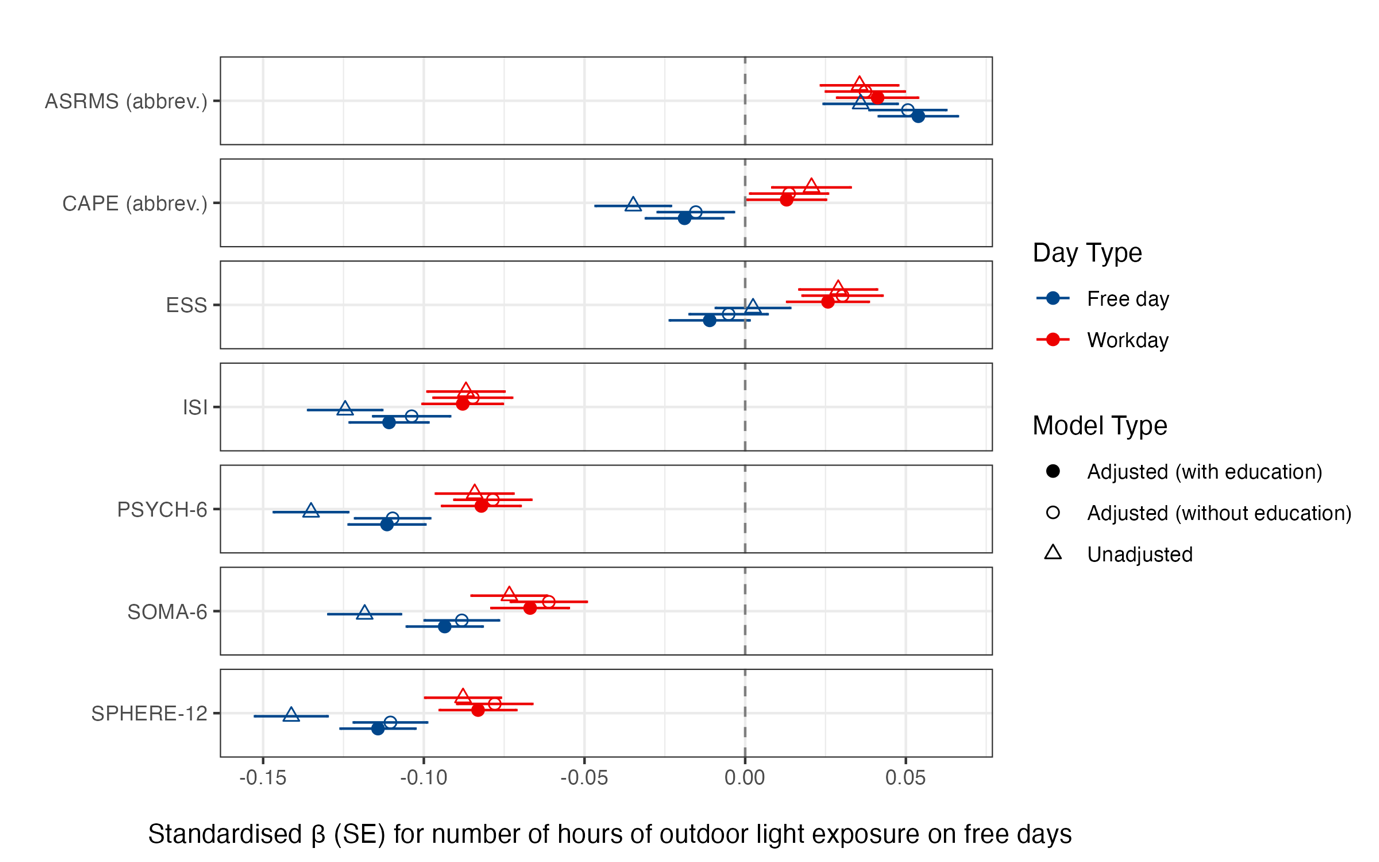


**Supplementary Figure 2. Summary of exploratory regression models (adjusted and unadjusted) examining associations among self-reported number of hours of outdoor light exposure on free days and workdays and seven dimensions of mental health across four age-groups.** Note: Note: Models are adjusted for age, sex, shift worker status, employment status, and educational attainment.


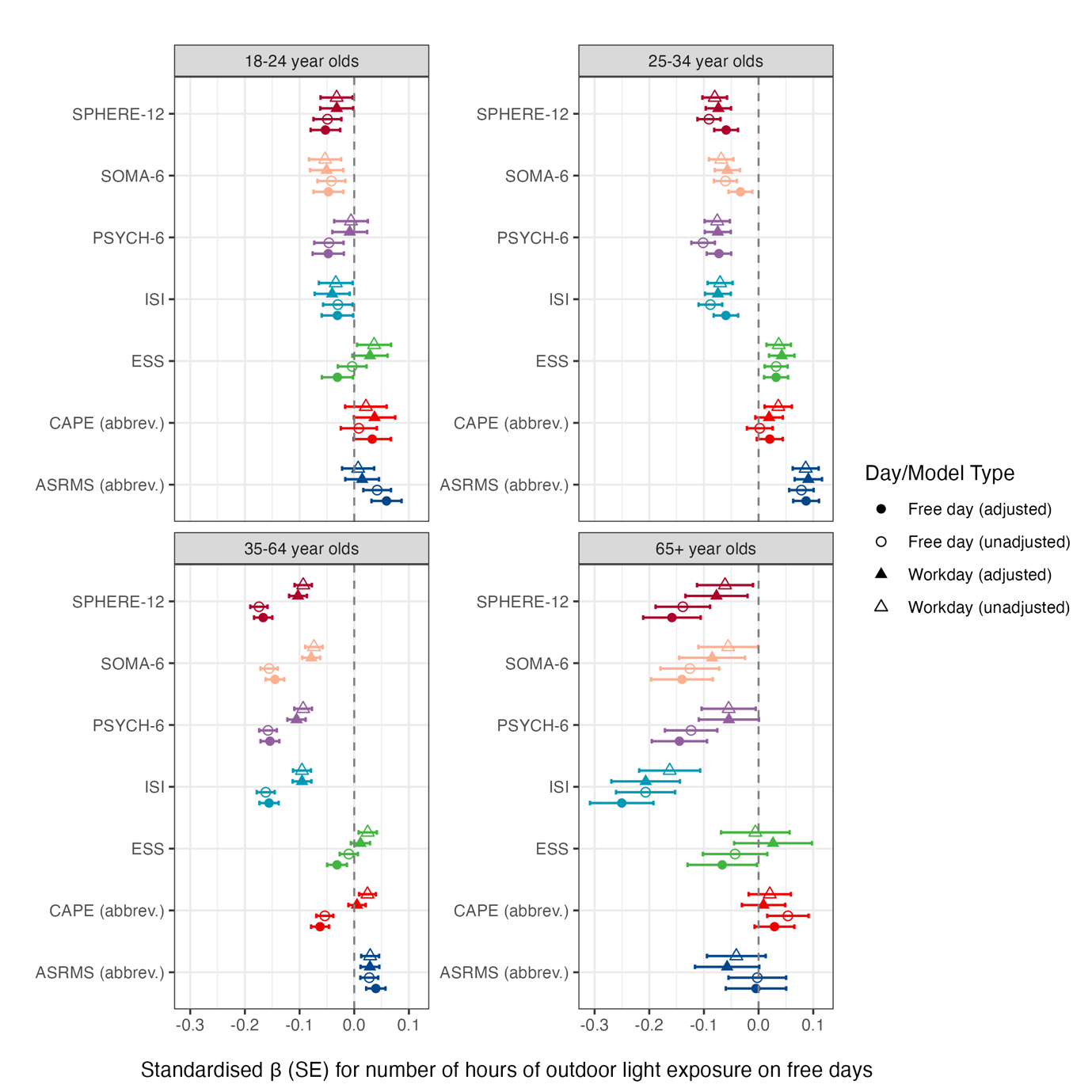


**Supplementary Figure 3. Primary analyses adjusting for chronotype.**


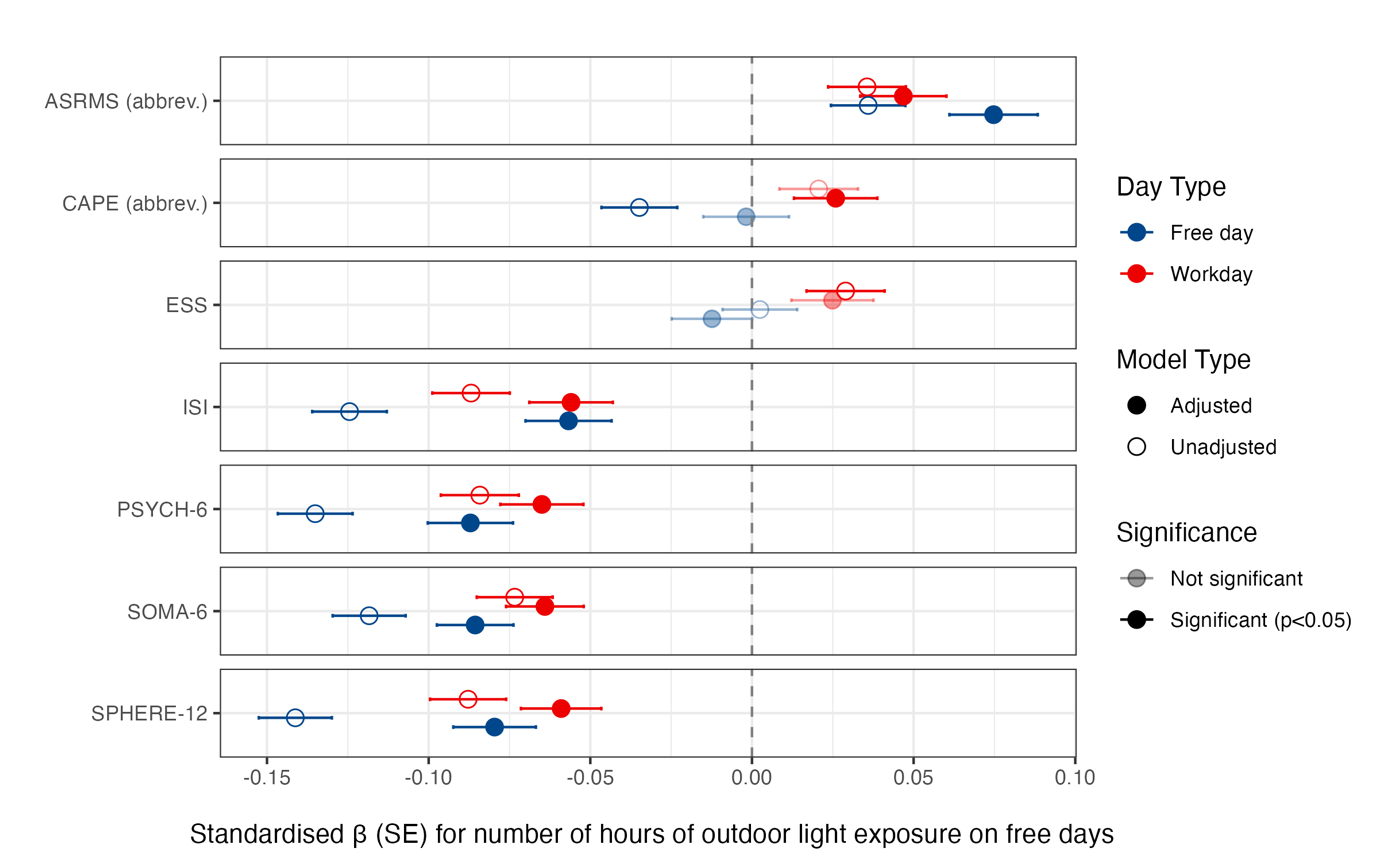


**Supplementary Figure 4. Primary analyses adjusting for insomnia (ISI).**

**
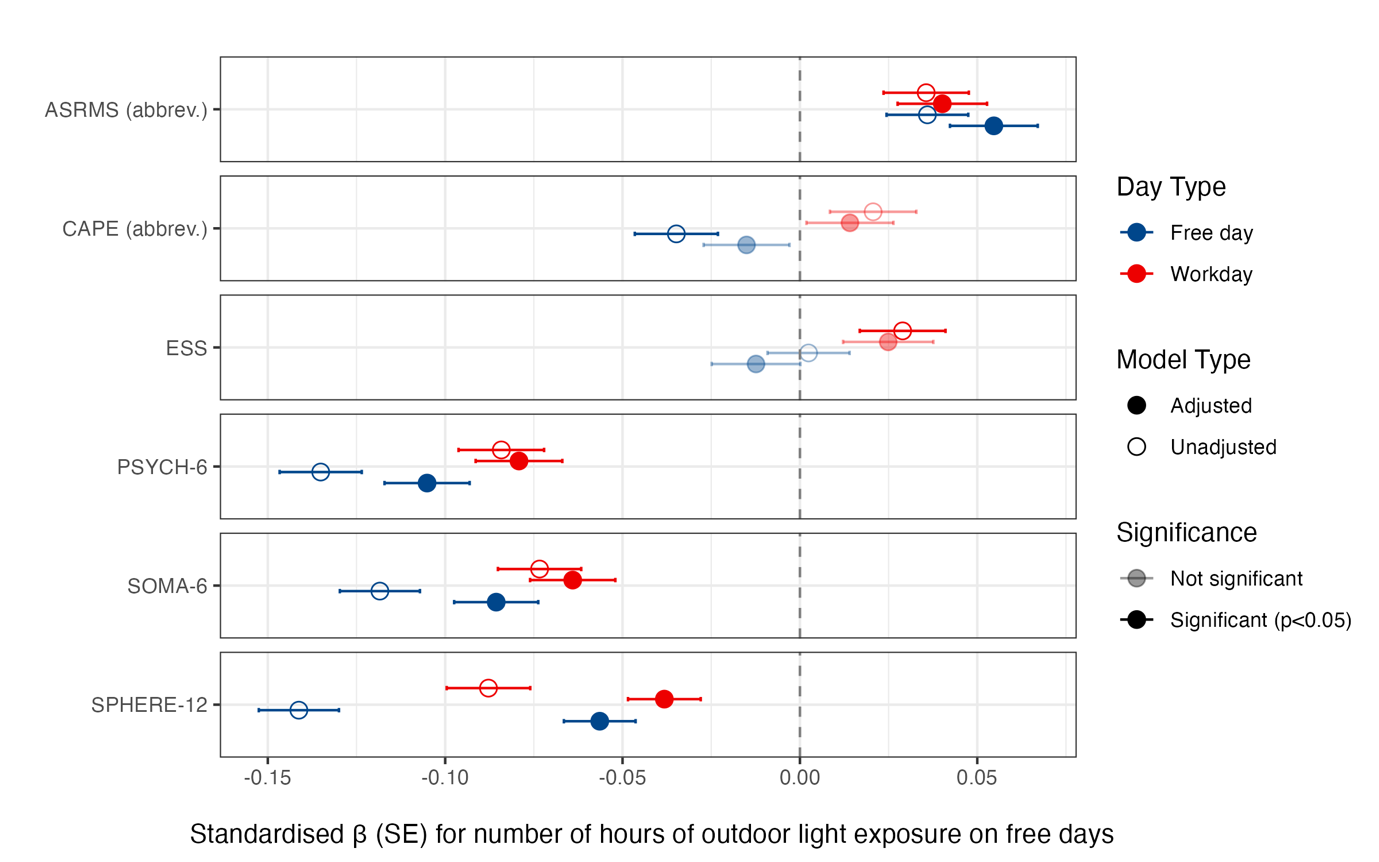
**

**Supplementary Figure 4. Age-stratified analyses adjusted for chronotype.**

**
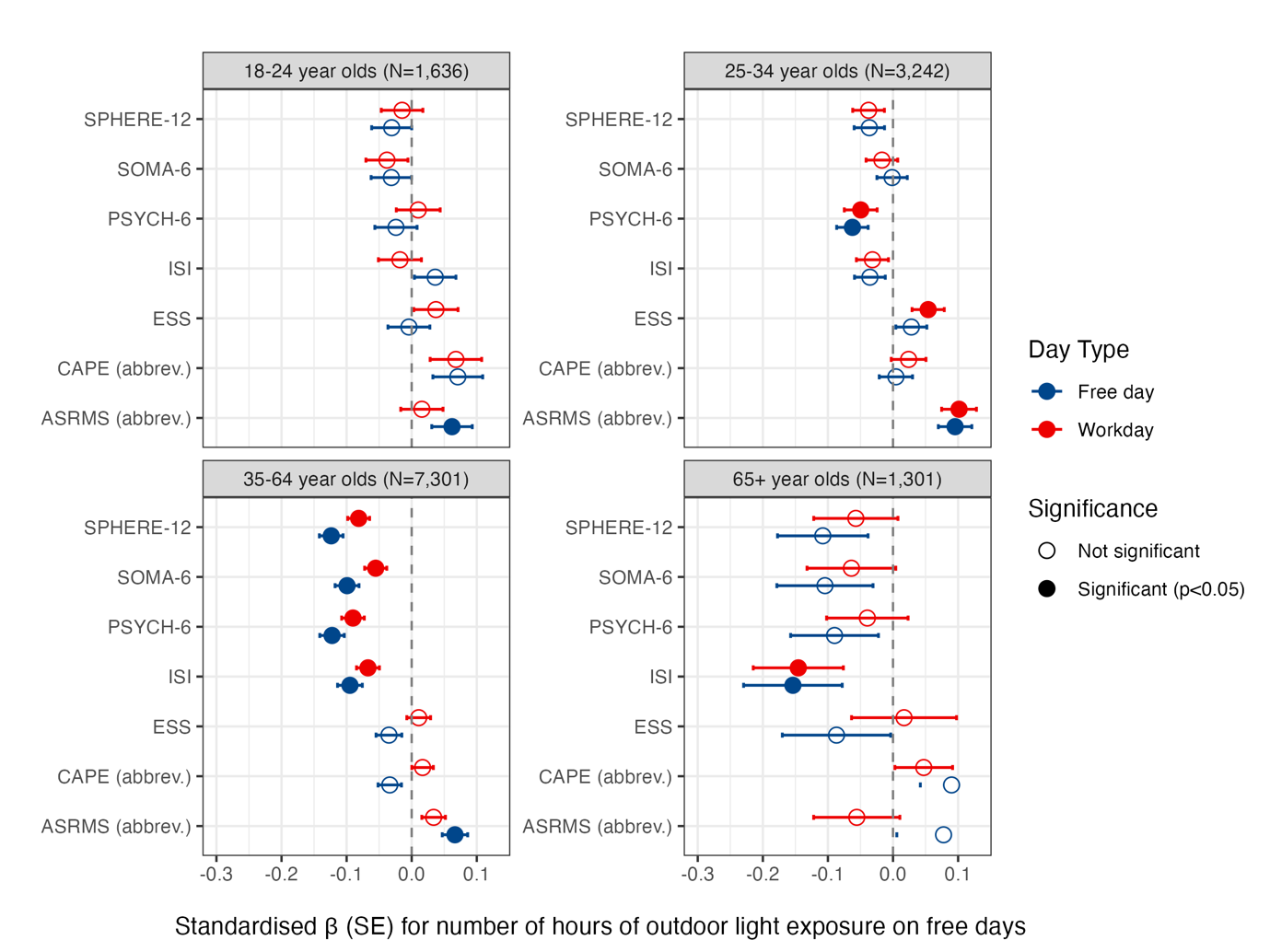
**

**Supplementary Figure 6. Age-stratified analyses adjusted for insomnia.**


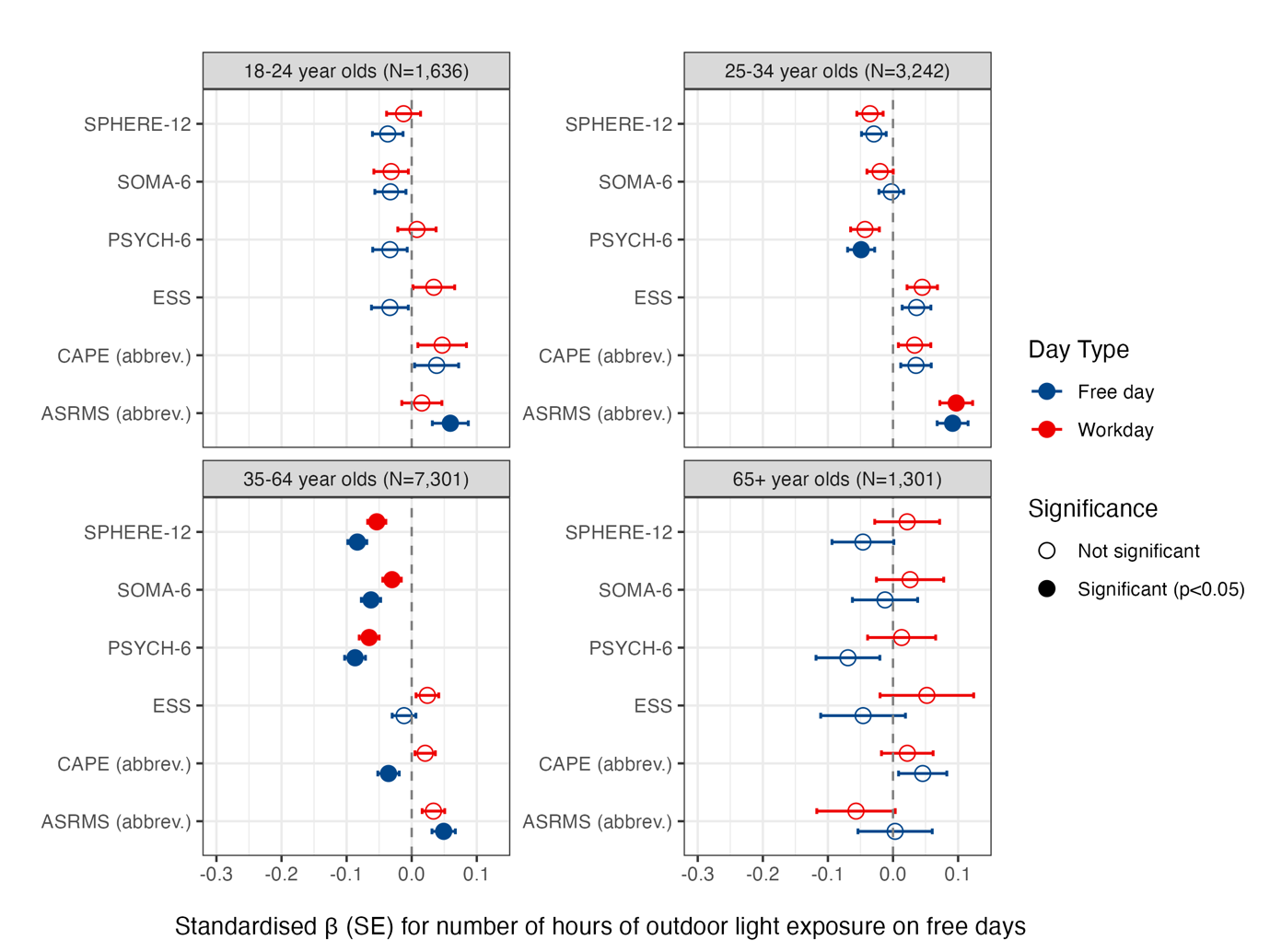


In the age-stratified analyses (Supplementary Figure 5), the patterns of association for each of the age groups was quite similar (albeit attenuated). Despite three previous associations no longer being significant at the nominal level (p<0.05), the general pattern of more daylight and workdays and free days and lower depressive symptoms (SPHERE-12, PSYCH-6, SOMA-6) remained similar.
